# Anti-inflammatory effect and action mechanisms of traditional herbal formula *Gamisoyo-san* in RAW 264.7 macrophages

**DOI:** 10.1186/s12906-016-1197-7

**Published:** 2016-07-15

**Authors:** Seong Eun Jin, Ohn Soon Kim, Sae-Rom Yoo, Chang-Seob Seo, Yeji Kim, Hyeun-Kyoo Shin, Soo-Jin Jeong

**Affiliations:** K-herb Research Center, Korea Institute of Oriental Medicine, Daejeon, 34054 Republic of Korea; KM Convergence Research Division, Korea Institute of Oriental Medicine, 1672 Yuseong-daero, Yuseong-gu, Daejeon, 34054 Republic of Korea; Mucosal Immunology Laboratory, Department of Convergence Medicine, University of Ulsan College of Medicine/Asan Medical Center, Seoul, 05505 Republic of Korea; Korean Medicine Life Science, University of Science & Technology, Daejeon, 34113 Republic of Korea

**Keywords:** *Gamisoyo-san*, Inflammatory mediator, Cytokine, Mitogen-activated protein kinases (MAPKs), Nuclear transcription factor kappa B (NF-κB)

## Abstract

**Background:**

*Gamisoyo-san* (GMSYS) is a traditional herbal formula used to treat insomnia, dysmenorrhea, and infertility in Korea. The purpose of this study was to investigate the anti-inflammatory effect and action mechanisms of GMSYS in lipopolysaccharide (LPS)-stimulated RAW 264.7 macrophages.

**Methods:**

The anti-inflammatory effects of GMSYS were investigated using nitric oxide (NO) assay and ELISAs for prostaglandin E_2_ (PGE_2_), tumor necrosis factor-α (TNF-α), and interleukin-6 (IL-6). The anti-inflammatory action mechanisms of GMSYS were evaluated using Western blotting for inducible nitric oxide synthase (iNOS), cyclooxygenase-2 (COX-2), and activation of nuclear transcription factor kappa B (NF-κB) and mitogen-activated protein kinases (MAPKs).

**Results:**

GMSYS significantly inhibited the LPS-induced production of NO, PGE_2_, TNF-α, and IL-6 compared with the vehicle-treated cells. GMSYS consistently downregulated the expression of iNOS and COX-2 mRNA induced by LPS. In addition, pretreatment with GMSYS suppressed the LPS-induced activation of NF-κB and MAPKs such as p38, extracellular signal-regulated kinase (ERK), and c-Jun N-terminal kinase (JNK).

**Conclusions:**

Our results indicate that the anti-inflammatory effects of GMSYS in RAW 264.7 macrophages are associated with inhibition of the release of inflammatory mediators and cytokines through the suppression of MAPK and NF-κB activation. These findings suggest that GMSYS may be a useful therapeutic candidate for the prevention or treatment of inflammatory diseases.

## Background

Inflammation is a complex process regulated by a cascade of inflammatory mediators, including nitric oxide (NO), prostaglandin E_2_ (PGE_2_), and cytokines, including tumor necrosis factor-α (TNF-α) and interleukin-6 (IL-6), that are produced by activated macrophages [1, 2].

NO is generated from l-arginine by nitric oxide synthase (NOS) and plays an important role in the regulation of physiological responses [3]. Among the isoforms of NOS, the expression of inducible NOS (iNOS) is specifically stimulated by cytokines and bacterial lipopolysaccharide (LPS) [4]. Prostaglandins are key inflammatory mediators that are produced from arachidonic acid by cyclooxygenase (COX). Among the isoforms of COX, COX-2 is induced after exposure to specific stimulants including cytokines and LPS. The induction of COX-2 produces a large amount of PGE_2_ that causes inflammatory reactions [5]. These inflammatory mediators and cytokines are critically associated with pain, fever, edema, and recruitment of additional immune cells to the site of inflammation [6]. However, overproduction of inflammatory mediators and cytokines is associated with tissue damage [7]. Therefore, inhibiting the release of inflammatory mediators and cytokines could be beneficial in attenuating the damage caused by inflammatory diseases.

Mitogen-activated protein kinases (MAPKs) such as p38, extracellular signal-regulated kinase (ERK), and c-Jun N-terminal kinase (JNK) regulate inflammatory and immune responses, and their signaling pathways are involved in LPS-induced iNOS and COX-2 expression in macrophages [8, 9]. In addition, nuclear transcription factor kappa B (NF-κB) is crucial during inflammation [10]. The regulatory free NF-κB translocates to the nucleus during inflammation, where it binds to κB-binding sites in the promotor regions of target genes and induces the transcription of inflammatory mediators and cytokines such as iNOS, COX-2, TNF-α, and IL-6 [11]. Thus, there may be an opportunity to improve the development of the inflammatory response by regulating the activation of the MAPK and NF-κB pathways.

In Korea, *Gamisoyo-san* (GMSYS) has been widely used for the treatment of dysmenorrhea, insomnia, and anxiety [12]. GMSYS is effective for treatment of sleep disturbances, headache, dizziness in postmenopausal women, depressive symptoms in premenstrual dysphoric disorder, and for tardive dyskinesia related to the use of antipsychotic drugs [13–15]. In addition, GMSYS has been reported to exert antistress, antidepressive, and antioxidant activities [12, 16–20]. Despite previous studies, there have been no reports on the effects and molecular mechanisms of GMSYS on inflammatory responses.

In the present study, we investigated the effect of GMSYS on the expression and release of inflammatory mediators and cytokines including NO, iNOS, PGE_2_, COX-2, TNF-α, and IL-6 from LPS-stimulated RAW 264.7 macrophages. In addition, we studied the molecular mechanisms possibly involved in the regulation of inflammatory responses by GMSYS.

## Methods

### Preparation of GMSYS water extract

The 12 raw herbal medicines composing the GMSYS formula were purchased from a traditional herb market, Kwangmyungdang Medicinal Herbs (Ulsan, Republic of Korea). The 12 herbal medicines were authenticated by an expert taxonomist, Prof. Je-Hyun Lee, Dongguk University, Gyeongju, Republic of Korea. Voucher specimens were deposited at the K-herb Research Center, Korea Institute of Oriental Medicine (2012–KE45–1 ~ KE45–11). To obtain a water decoction of GMSYS, the 12 herbal medicines were mixed as shown in Table 1 (total weight = 5.0 kg, about 141.8 times the composition of a single dose) and extracted in distilled water at 100 °C for 2 h under pressure (98 kPa) using an electric extractor (COSMOS-660; Kyungseo Machine Co., Incheon, Korea). The extract was filtered using a standard sieve (No. 270, 53 μm; Chung Gye Sang Gong Sa, Seoul, Korea) and lyophilized to give a powder sample. The yield of GMSYS extract was about 19.4 % (970.4 g).

**Table 1:** Composition of GMSYS

Latin name	Scientific name	Amount (g)	Origin
Paeoniae Radix	*Paeonia lactiflora* Pallas	4.500	Uiseong, Korea
Atractylodis Rhizoma Alba	*Atractylodes macrocephala* Koidzumi	4.500	China
Anemarrhenae Rhizoma	*Anemarrhena asphodeloides* Bunge	3.750	Kangjin, Korea
Lycii Radicis Cortex	*Lycium chinense* Miller	3.750	China
Angelicae Gigantis Radix	*Angelica gigas* Nakai	3.750	Bonghwa, Korea
Poria Sclerotium	*Poria cocos* Wolf	3.000	Pyeongchang, Korea
Liriope Tuber	*Liriope platyphylla* Wang et Tang	3.000	Miryang, Korea
Rehmanniae Radix Crudus	*Rehmannia glutinosa* Liboschitz var. *purpurea* Makino	3.000	Gunwi, Korea
Gardeniae Fructus	*Gardenia jasminoides* Ellis	1.875	Gurye, Korea
Phellodendri Cortex	*Phellodendron amurense* Ruprecht	1.875	China
Platycodi Radix	*Platycodon grandiflorum* A. De Candolle	1.125	Muju, Korea
Glycyrrhizae Radix et Rhizoma	*Glycyrrhiza uralensis* Fischer	1.125	China
Total		35.250	

### High-performance liquid chromatography (HPLC) analysis of GMSYS

Quantitative analysis of the GMSYS sample was performed using an LC-20A Prominence HPLC system (Shimadzu Corp., Kyoto, Japan) equipped with a solvent delivery unit, an on-line degasser, a column oven, an autosampler, and a photo diode array (PDA) detector. Data were acquired and processed using LabSolution software (version 5.54, SP3; Shimadzu Corp.). Separation was achieved on a SunFire C_18_ analytical column (250 × 4.6 mm; particle size 5 μm, Waters, Milford, MA, USA) as the stationary phase at a column temperature set to 40 °C. The mobile phases consisted of 0.1 % (v/v) formic acid in water (A) and 0.1 % (v/v) formic acid in acetonitrile (B). The gradient sequence and elution conditions were as follows: 5–60 % B for 0–30 min, 60–100 % B for 30–40 min, 100 % B for 40–45 min, 100–10 % B for 45–50 min, with a reequilibrium time of 10 min. The flow-rate was 1.0 mL/min, and the sample injection volume was 10 μL. For HPLC analysis, 200 mg of lyophilized GMSYS extract was dissolved in 20 mL of distilled water and then the solution diluted 10-fold for quantitative analysis of geniposide and paeoniflorin. Samples were filtered through a SmartPor GHP 0.2 μm syringe filter (Woongki Science, Seoul, Korea) before application onto the HPLC column.

### Cell culture

The murine macrophage cell line, RAW 264.7, was obtained from the American Type Culture Collection (Rockville, MD). The cells were cultured in Dulbecco’s modified Eagle’s medium (Gibco Inc., Grand Island, NY) supplemented with 5.5 % heat-inactivated fetal bovine serum (Gibco Inc.), penicillin (100 U/mL), and streptomycin (100 μg/mL) in a 5 % CO_2_ incubator at 37 °C.

### Cytotoxicity assay

A cell viability assay was performed to determine the cytotoxicity of GMSYS using a Cell Counting Kit-8 (CCK-8; Dojindo Laboratories, Kumamoto, Japan). RAW 264.7 macrophages were plated into a 96-well microplate at 3 × 10^3^ cells/well and treated with various concentrations of GMSYS for 24 h. After incubation with CCK-8 reagent for 4 h, optical density (OD) at 450 nm was measured by using a Benchmark plus microplate reader (Bio-Rad Laboratories, Hercules, CA). The cell viability was calculated using the following equation:

$$ \mathrm{Cell}\kern0.5em \mathrm{viability}\kern0.5em \left(\%\right)=\frac{\mathrm{Mean}\kern0.5em \mathrm{O}\mathrm{D}\kern0.5em \mathrm{in}\kern0.5em \mathrm{GMSYS}\kern0.5em \mathrm{treated}\kern0.5em \mathrm{cells}}{\mathrm{Mean}\kern0.5em \mathrm{O}\mathrm{D}\kern0.5em \mathrm{in}\kern0.5em \mathrm{untreated}\kern0.5em \mathrm{cells}}\times 100 $$

### NO assay

RAW 264.7 macrophages were pretreated with various concentrations of GMSYS (0, 250, 500, or 1000 μg/mL) for 4 h and stimulated with LPS (1 μg/mL) for an additional 20 h. NO synthesis was determined by measuring the accumulation of nitrite in the culture supernatant using a Griess Reagent System (Promega, Madison, WI). Briefly, an equal volume of supernatant and sulfanilamide solution was mixed and incubated for 10 min at room temperature, and then naphthylethylenediamine dihydrochloride solution was added. The mixture was incubated for an additional 5 min, and its absorbance was measured at 540 nm using a Benchmark plus microplate reader (Bio-Rad Laboratories). The nitrite concentration in the supernatants was determined from a standard curve generated with sodium nitrite.

### ELISAs for PGE_2_, TNF-α, and IL-6

Production of PGE_2_, TNF-α, and IL-6 in the LPS-stimulated RAW 264.7 macrophage culture supernatants was measured using commercial ELISA kits from Cayman Chemical Co. (Ann Arbor, MI), Invitrogen (Carlsbad, CA), and BD Biosciences (Mountain View, CA), respectively.

### RNA extraction and quantitative real-time polymerase chain reaction (RT-qPCR)

Total RNA was extracted using Trizol reagent (Invitrogen Life Sciences, Carlsbad, CA) according to the manufacturer’s instructions. Complementary DNA (cDNA) was synthesized from 1 μg of total RNA using an iScript cDNA synthesis kit (Bio-Rad Laboratories). RT-qPCR was performed by using a Rotor Gene Q system (Qiagen, Hilden, Germany) and a reaction mixture that consisted of SYBR Green 2 × PCR Master Mix, cDNA template, and forward and reverse primers. The PCR protocol consisted of 35 cycles of denaturation at 95 °C for 15 sec, followed by 55 °C for 30 s to allow for extension and amplification of the target sequence. The relative levels of iNOS and COX-2 mRNA expression were normalized to that of glyceraldehyde 3-phosphate dehydrogenase (GAPDH) using the 2-ΔΔCT method. The primer sequences used in this study are shown in Table 2.

**Table 2:** Nucleotide sequences of primers used in real-time RT-PCR

Gene	Forward	Reverse	Accession no.
iNOS	AAGGTCTACGTTCAGGACATC	AGAAATAGTCTTCCACCTGCT	NM_010927
COX-2	TTCCTCTACATAAGCCAGTGA	TCCACATTACATGCTCCTATC	NM_011198
GAPDH	TGTGTCCGTCGTGGATCTGA	CCTGCTTCACCACCTTCTTGA	NM_008084

### Western blotting

RAW 264.7 macrophages were pretreated with GMSYS (0, 250, 500, or 1000 μg/mL) for 4 h and then treated with or without LPS (1 μg/mL) for 15 min (to detect phosphorylation of protein), or for 20 h (to detect total protein). Whole cell extract (WCE) was prepared by suspending the macrophages in an extraction lysis buffer (Sigma-Aldrich, St. Louis, MO) containing protease inhibitor cocktail (Roche Applied Science, Indianapolis, IN). Nuclear extract (NE) was isolated using NE-PER Nuclear and Cytoplasmic Extraction reagents (Thermo Fisher Scientific, Rockford, IL) according to the manufacturer’s protocol. The protein concentration in the cell extracts was determined using a Bio-Rad Protein Assay Reagent (Bio-Rad Laboratories). Equal amounts of protein (30 μg) were resolved by 4–20 % sodium dodecyl sulfate-polyacrylamide gel electrophoresis (SDS-PAGE) and transferred to a polyvinylidene fluoride (PVDF) membrane. The membrane was incubated with a solution of 5 % skim milk in Tris-buffered saline containing Tween 20 (TBST) to block nonspecific binding sites, followed by overnight incubation at 4 °C with an appropriate primary antibodies; anti-phospho-p38 MAPK, anti-phospho-ERK, anti-phospho-JNK (Cell Signaling, Danvers, MA), anti-NF-κB p65, anti-β-actin, anti-α-tubulin and anti-Lamin B (Santa Cruz Biotechnology, Dallas, TX). The membranes were washed three times with TBST, and then incubated with a corresponding horseradish peroxidase-conjugated secondary antibody (Jackson ImmunoResearch, West Grove, PA) for 1 h at room temperature. The membranes were washed three times with TBST again and then immunereactivity visualized using an enhanced chemiluminescence (ECL) (Thermo Fisher Scientific). Images were captured using Chemi-Doc (Bio-Rad Laboratories).

### Statistical analyses

The data are expressed as the mean ± SEM. Data were analyzed using a one-way analysis of variance and Dunnett’s multiple comparisons test. *P* < 0.05 was considered to be significant.

## Results

### Identification and quantification of the marker components of GMSYS

**Fig. 1:**
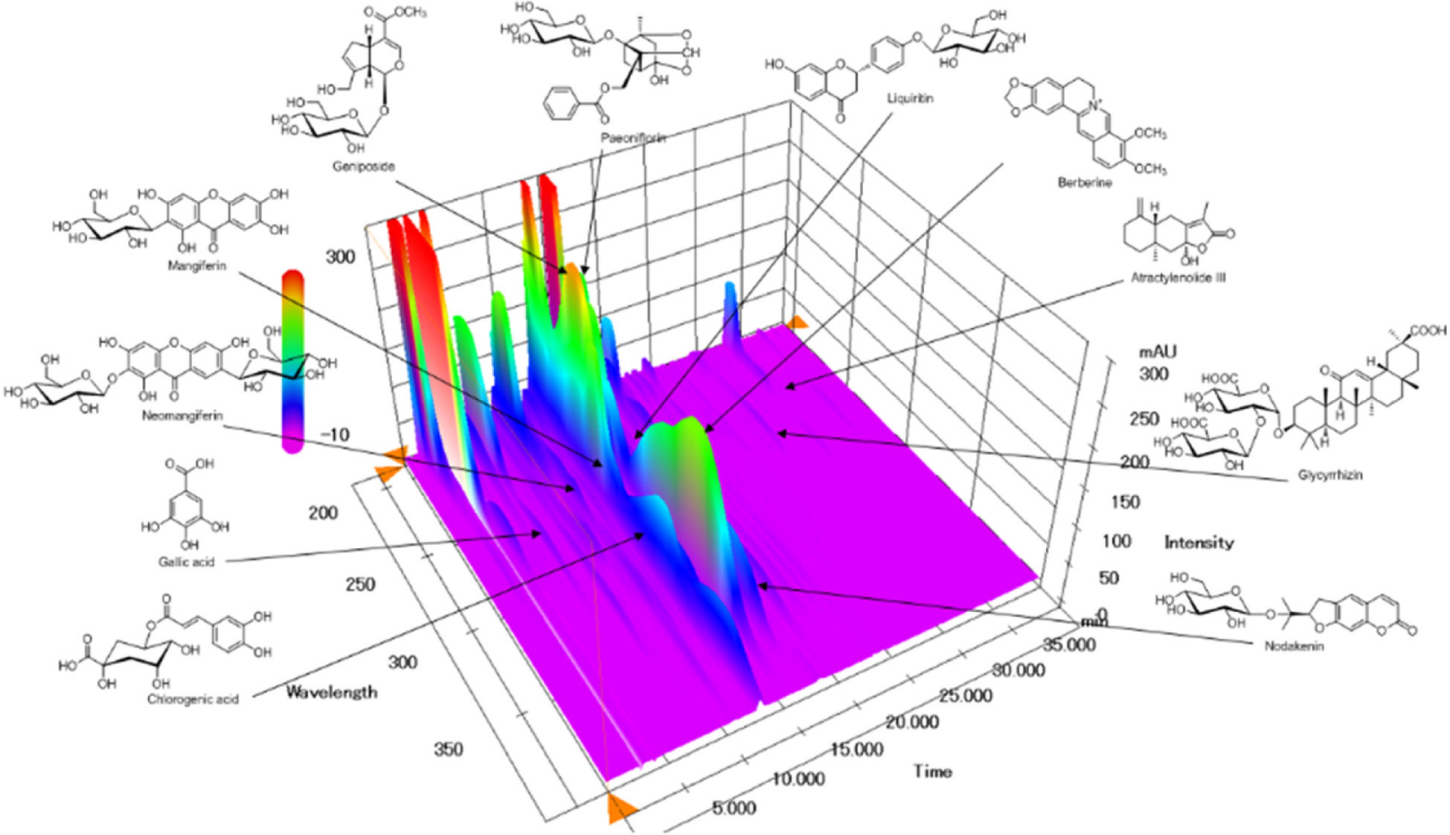
Three-dimensional chromatogram of GMSYS by HPLC-PDA. The retention times of 11 marker compounds—gallic acid, neomangiferin, chlorogenic acid, mangiferin, geniposide, paeoniflorin, berberine, liquiritin, nodakenin, glycyrrhizin, and atractylenolide III—were approximately 6.32, 10.82, 12.63, 13.04, 13.53, 15.17, 15.35, 16.61, 17.27, 29.86, and 34.00 min, respectively

We selected 11 components—including gallic acid, neomangiferin, chlorogenic acid, mangiferin, geniposide, paeoniflorin, berberine, liquiritin, nodakenin, glycyrrhizin, and atractylenolide III—as marker compounds of GMSYS. Calibration curves for the 11 marker components showed good linearity with correlation coefficients (*r*^2^) ≥ 0.9996 in their various concentration ranges. Using optimized chromatography conditions, a three-dimensional chromatogram was obtained using HPLC–PDA detector, and the 11 compounds were eluted within 35 min (Fig. 1). The concentrations of gallic acid, neomangiferin, chlorogenic acid, mangiferin, geniposide, paeoniflorin, berberine, liquiritin, nodakenin, glycyrrhizin, and atractylenolide III were 0.58 ± 0.01, 0.45 ± 0.01, 0.90 ± 0.01, 1.99 ± 0.01, 8.37 ± 0.05, 8.91 ± 0.09, 2.01 ± 0.001, 0.94 ± 0.002, 1.43 ± 0.01, 0.81 ± 0.01, and 0.04 ± 0.001 mg/g, respectively.

### Cytotoxicity of GMSYS in RAW 264.7 macrophages

**Fig. 2:**
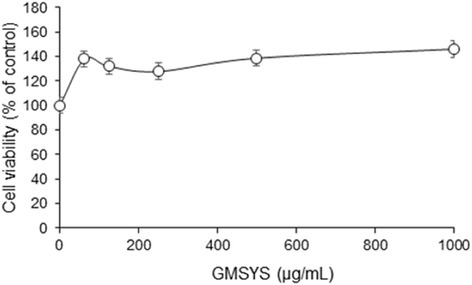
Effect of GMSYS on the cell viability in RAW 264.7 macrophages. The macrophages were treated with various concentrations of GMSYS (0, 62.5, 125, 250, 500, or 1000 μg/mL) for 24 h. Cell viability was measured using a CCK assay

To determine the cytotoxicity of GMSYS, RAW 264.7 macrophages were treated with GMSYS at various concentrations ranging from 62.25 to 1000 μg/mL for 24 h, and a viability assay was conducted. As shown in Fig. 2, GMSYS did not produce any significant cytotoxicity up to 1000 μg/mL. Subsequent experiments were performed at nontoxic concentrations.

### Effect of GMSYS on NO production and iNOS expression in LPS-stimulated RAW 264.7 macrophages

**Fig. 3:**
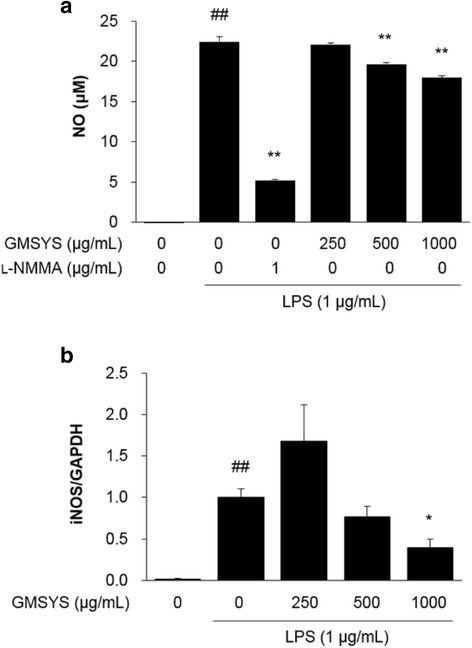
Effect of GMSYS on the production of NO and expression of iNOS in LPS-stimulated RAW 264.7 macrophages. The macrophages were pretreated with GMSYS for 4 h and then treated with LPS (1 μg/mL) for 20 h. **a** Collected supernatants were reacted with Griess reagent, and absorbance was measured at 540 nm. **b** Total RNA was isolated from the cell pellets and subjected to RT-qPCR to detect the expression of iNOS mRNA. Levels of iNOS mRNA expression were normalized to the expression of GAPDH mRNA. Bar graphs represent the means from three independent experiments. ^##^ *P* < 0.01 *vs* vehicle-treated cells and ^*^ *P* < 0.05 or ^**^ *P* < 0.01 *vs* LPS-treated cells

The level of NO in culture supernatant was measured using a Griess reaction. As shown in Fig. 3a, LPS treatment significantly increased the level of NO compared with vehicle-treated macrophages (*P* < 0.01). By contrast, LPS-induced NO production was markedly reduced in macrophages pretreated with GMSYS (500 or 1000 μg/mL). A positive control using *N*-methyl-l-arginine (l-NMMA, 100 μM) showed a significant decrease in LPS-induced NO production.

To investigate the cause of reduced NO production by GMSYS, the expression of iNOS by the macrophages was determined by RT-qPCR. LPS treatment of the macrophages significantly increased the expression of iNOS compared with vehicle-treated macrophages (*P* < 0.01). By contrast, GMSYS (500 or 1000 μg/mL) pretreatment significantly decreased the expression of LPS-induced iNOS (*P* < 0.01, Fig. 3b).

### Effect of GMSYS on PGE_2_ production and COX-2 expression in LPS-stimulated RAW 264.7 macrophages

**Fig. 4:**
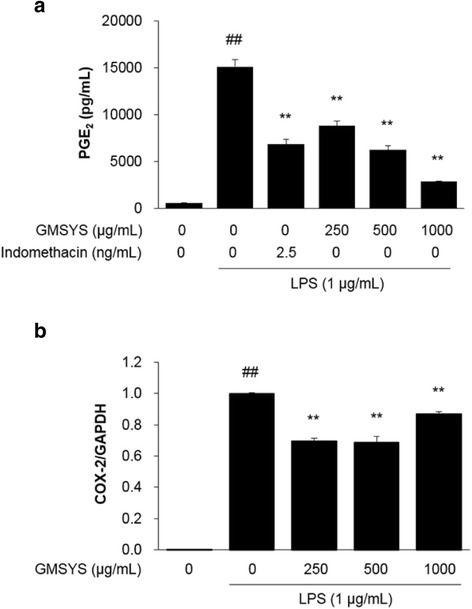
Effect of GMSYS on the production of PGE_2_ and expression of COX-2 in LPS-stimulated RAW 264.7 macrophages. The macrophages were pretreated with GMSYS for 4 h and then treated with LPS (1 μg/mL) for 20 h. **a** PGE_2_ levels in supernatants were measures by ELISA. **b** Total RNA was isolated from the cell pellets and subjected to RT-qPCR to detect the expression of COX-2 mRNA. Levels of COX-2 mRNA expression were normalized to the expression of GAPDH mRNA. Bar graphs represent the means from three independent experiments. ^##^ *P* < 0.01 *vs* vehicle-treated cells and ^**^ *P* < 0.01 *vs* LPS-treated cells

As shown in Fig. 4a, LPS-treated macrophages produced significantly increased levels of PGE_2_ compared with the vehicle-treated macrophages. By contrast, pretreatment with GMSYS (250, 500, or 1000 μg/mL) significantly decreased the level of PGE_2_ produced by the LPS-stimulated macrophages compared with macrophages treated with LPS alone (*P* < 0.01) (Fig. 3b). Indomethacin (2.5 ng/mL) was used as a positive control and markedly decreased PGE_2_ production by the stimulated macrophages.

To investigate whether the inhibitory effect of GMSYS on PGE_2_ production was related to the expression of COX-2, we measured the COX-2 mRNA expression using RT-qPCR. As shown in Fig. 4b, the expression of COX-2 was significantly induced by LPS stimulation (*P* < 0.01). By contrast, pretreatment of macrophages with GMSYS (250, 500, or 1000 μg/mL) markedly inhibited their LPS-induced COX-2 expression (*P* < 0.05, Fig. 4b).

### Effect of GMSYS on the production of inflammatory cytokines in LPS-stimulated RAW 264.7 macrophages

**Fig. 5:**
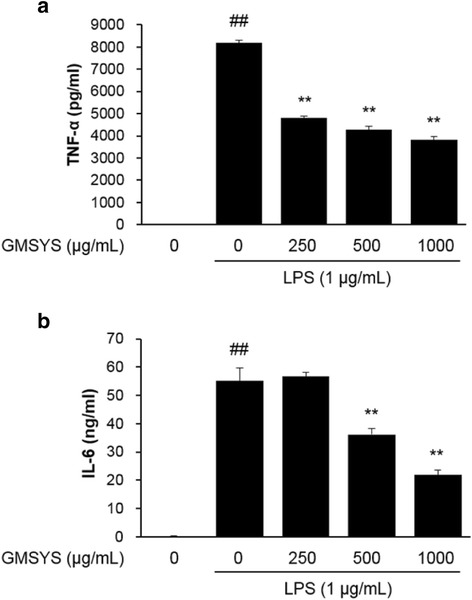
Effect of GMSYS on the production of TNF-α and IL-6 in LPS-stimulated RAW 264.7 macrophages. The macrophages were pretreated with GMSYS (0, 250, 500, or 1000 μg/mL) for 4 h and then treated with LPS (1 μg/mL) for 20 h. ELISAs were used to determined levels of (**a**) TNF-α and (**b**) IL-6 in collected supernatants. Bar graphs represent the means from three independent experiments. ^##^ *P* < 0.01 *vs* vehicle-treated cells and ^**^ *P* < 0.01 *vs* LPS-treated cells

To determine the effect of GMSYS on the production of proinflammatory cytokines TNF-α and IL-6, RAW 264.7 macrophages were pretreated with various concentrations of GMSYS (0, 250, 500, or 1000 μg/mL) for 4 h and then stimulated with LPS (1 μg/mL) for 20 h. As shown in Fig. 5, the levels of TNF-α and IL-6 were significantly increased in LPS-stimulated RAW 264.7 macrophages compared with vehicle-treated macrophages (*P* < 0.01). By contrast, pretreatment of the macrophages with GMSYS (250, 500, or 1000 μg/mL) significantly decreased the levels of TNF-α in a dose-dependent manner (*P* < 0.01) (Fig. 5a). LPS-induced IL-6 levels were also reduced by pretreatment of the macrophages with GMSYS (500 or 1000 μg/mL) (*P* < 0.01, Fig. 5b).

### Effect of GMSYS on MAPK phosphorylation in LPS-stimulated RAW 264.7 macrophages

**Fig. 6:**
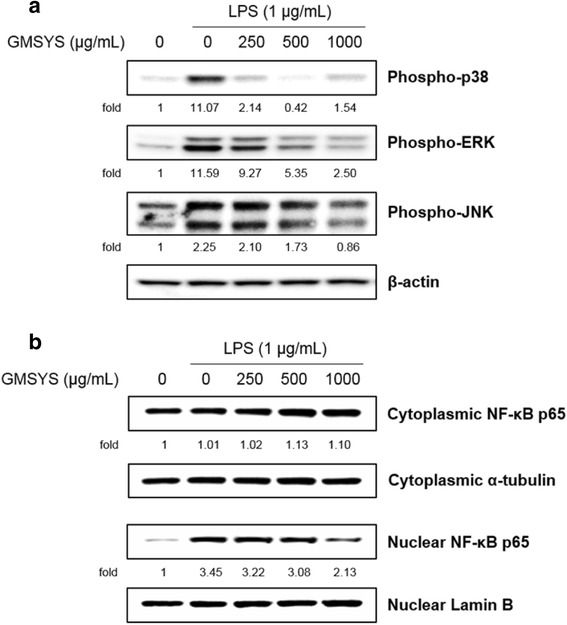
Effect of GMSYS on the phosphorylation of MAPKs and activation of NF-κB in LPS-stimulated RAW 264.7 macrophages. **a** The macrophages were pretreated with GMSYS (0, 250, 500, or 1000 μg/mL) for 2 h and then treated with LPS (1 μg/mL) for 15 min. Immunoblotting was used to detect the phosphorylation of p38 MAPK, ERK, and JNK in lysates prepared from the macrophages. **b** The macrophages were pretreated with GMSYS (0, 250, 500, or 1000 μg/mL) for 2 h and then treated with LPS (1 μg/mL) for 1 h. Immunoblotting of cytoplasmic and nuclear extracts prepared from the macrophages was used to detect the translocation of and subjected to immunoblotting for NF-κB p65. α-Tubulin and Lamin B were used as an internal control for cytoplasm and nucleus, respectively

To clarify the molecular mechanism of the anti-inflammatory effects of GMSYS, we analyzed the phosphorylation of p38, ERK, and JNK by Western blotting. As shown in Fig. 6a, GMSYS (250, 500, or 1000 μg/mL) decreased the expression of phosphorylated p38 in LPS-stimulated RAW 264.7 macrophages. In addition, the phosphorylation of ERK and JNK was also reduced by pretreatment of GMSYS (500 or 1000 μg/mL).

### Effect of GMSYS on NF-κB activation in LPS-stimulated RAW 264.7 macrophages

To investigate the mechanism by which GMSYS reduces LPS-induced inflammatory responses, the nuclear translocation of NF-κB p65 was assessed by Western blotting. As shown in Fig. 6b, nuclear NF-κB p65 was markedly increased in the nucleus of LPS-treated macrophages compared with the vehicle alone. By contrast, pretreatment with GMSYS reduced the nuclear translocation of NF-κB p65 in LPS-treated macrophages compared with those without such pretreatment.

## Discussion

GMSYS, a traditional herbal formula comprising 12 different herbal medicines—Paeoniae Radix, Atractylodis Rhizoma Alba, Anemarrhenae Rhizoma, Lycii Radicis Cortex, Angelicae Gigantis Radix, Poria Sclerotium, Liriope Tuber, Rehmanniae Radix Crudus, Gardeniae Fructus, Phellodendri Cortex, Platycodi Radix, and Glycyrrhizae Radix et Rhizoma—has been used in Korea to treat dysmenorrhea, insomnia, and anxiety [12]. Eleven of these 12 herbal medicines exhibit anti-inflammatory properties; namely, Paeoniae Radix [21], Atractylodis Rhizoma Alba [22], Anemarrhenae Rhizoma [23], Lycii Radicis Cortex [24], Angelicae Gigantis Radix [25], Poria Sclerotium [26], Rehmanniae Radix Crudus [27], Gardeniae Fructus [28], Phellodendri Cortex [29], Platycodi Radix [30], and Glycyrrhizae Radix et Rhizoma [31].

In the present study, we analyzed 11 marker compounds found in an GMSYS—including gallic acid, neomangiferin, chlorogenic acid, mangiferin, geniposide, paeoniflorin, berberine, liquiritin, nodakenin, glycyrrhizin, and atractylenolide III—using HPLC-PDA detector. Nine of these compounds have anti-inflammatory effects; namely, gallic acid [32], chlorogenic acid [33], mangiferin [34], geniposide [35], paeoniflorin [36], berberine [37], nodakenin [38], glycyrrhizin [39], and atractylenolide III [40]. For these reasons, we predict that GMSYS, which contains these anti-inflammatory herbal medicines and components, has a preventive and therapeutic effect on inflammatory diseases. However, the anti-inflammatory effect and mechanisms of its action has not been thoroughly elucidated. Therefore, we sought to investigate the anti-inflammatory effect and molecular mechanisms of an extract of GMSYS in LPS-stimulated RAW 264.7 macrophages, to our knowledge for the first time. We found that GMSYS inhibited the release of inflammatory mediators such as NO and PGE_2_, and downregulated iNOS and COX-2 expression in LPS-stimulated macrophages. These findings suggest that GMSYS decreases NO and PGE_2_ levels by suppressing iNOS and COX-2 expression, respectively.

TNF-α and IL-6 have been implicated in autoimmune diseases including rheumatoid arthritis [41]. Therefore, the measurement of TNF-α and IL-6 production may suggest an anti-inflammatory effect. In the present study, we showed that GMSYS suppressed the production of TNF-α and IL-6 in LPS-stimulated RAW 264.7 macrophages.

MAPK represents a group of signaling molecules that appear to play critical roles in inflammatory processes. In particular, they control cellular responses to cytokines and play an important role in the modulation of NF-κB activity. LPS activates three molecules involved in MAPK cascades, p38, ERK, and JNK [42, 43]. To identify the signaling pathways involved in the GMSYS-mediated anti-inflammatory responses, we investigated the phosphorylation of these molecules by Western blotting. GMSYS diminished the phosphorylation of p38, ERK, and JNK in LPS-stimulated RAW 264.7 macrophages.

NF-κB plays an important role in various pathological states as a transcription factor for iNOS, TNF-α, and IL-1β, and is translocated into the nucleus by LPS stimulation [44]. In the present study, we found that GMSYS attenuated the nuclear translocation of p65 in LPS-stimulated RAW 264.7 macrophages. These findings suggest that GMSYS has an anti-inflammatory effect by inhibiting NF-κB activation.

Although synthetic drugs have been used for treatment of inflammatory diseases such as atherosclerosis, many of them exhibit varying degrees of adverse effects [45]. By contrast, an examination of herbal medicines may contribute to the discovery of novel drugs as potential anti-inflammatory agents with fewer side effects. Some traditional herbal formulas produce their anti-inflammatory effects by inhibiting the production of inflammatory mediators by blocking NF-kB activation in RAW 264.7 macrophages [46, 47]. These reports and our findings suggest that natural products including GMSYS could be used as anti-inflammatory agents with fewer adverse effects than synthetic drugs.

## Conclusions

In conclusion, the present study showed that GMSYS inhibits the production and expression of NO, iNOS, PGE_2_, COX-2, TNF-α, and IL-6 in LPS-stimulated RAW 264.7 macrophages. These effects of GMSYS are related to the suppression of MAPK and NF-κB activation. Our results suggest that GMSYS should be considered as a source of potent anti-inflammatory candidates for the treatment or prevention of inflammatory diseases.

## Abbreviations

CCK-8, cell counting kit-8; cDNA, complementary DNA; COX, cyclooxygenase; ECL, enhanced chemiluminescence; ERK, extracellular signal-regulated kinase; GAPDH, glyceraldehyde 3-phosphate dehydrogenase; GMSYS, *Gamisoyo-san*; IL-6, interleukin-6; iNOS, inducible nitric oxide synthase; JNK, c-Jun N-terminal kinase; l-NMMA, *N*-methyl-l-arginine; LPS, lipopolysaccharide; MAPK, mitogen-activated protein kinase; NE, nuclear extract; NF-κB, nuclear transcription factor kappa B; NO, nitric oxide; NOS, nitric oxide synthase; OD, optical density; PDA, photo diode array; PGE_2_, prostaglandin E_2_; PVDF, polyvinylidene fluoride; SDS-PAGE, sodium dodecyl sulfate-polyacrylamide gel electrophoresis; TBST, tris-buffered saline containing Tween 20; TNF-α, tumor necrosis factor-α; WCE, whole cell extract
